# Inflammatory and Bleeding Risks on Clinical Outcomes in Acute Coronary Syndrome Patients Undergoing Percutaneous Coronary Intervention

**DOI:** 10.1055/a-2531-3268

**Published:** 2025-03-13

**Authors:** Yixuan Duan, Miaohan Qiu, Kun Na, Daoshen Liu, Shangxun Zhou, Ying Xu, Zizhao Qi, Haiwei Liu, Kai Xu, Xiaozeng Wang, Jing Li, Yi Li, Yaling Han

**Affiliations:** 1 Department of Cardiology, State Key Laboratory of Frigid Zone Cardiovascular Disease, General Hospital of Northern Theater Command, Shenyang, China; 2 The Department of Cardiology, Air Force Medical University, Xijing Hospital, Xi'an, Shaanxi, China

**Keywords:** acute coronary syndrome, high bleeding risk, high-sensitivity C-reactive protein, inflammation

## Abstract

**Objective:**

This study aimed to evaluate the impact of systemic inflammation burden using high-sensitivity C-reactive protein (hsCRP) and long-term prognosis in acute coronary syndrome (ACS) patients undergoing percutaneous coronary intervention (PCI) stratified by bleeding risk status.

**Methods:**

Consecutive patients admitted for ACS and who received PCI between March 2016 and March 2022 were enrolled in the analysis. Elevated systemic inflammation was defined as hsCRP >2 mg/L, and high bleeding risk (HBR) was defined the Academic Research Consortium (ARC)-HBR criteria. The primary outcome was ischemic events at 12 months, composed of cardiac death, myocardial infarction, and/or stroke. The main secondary outcomes included all-cause death, and Bleeding Academic Research Consortium (BARC) types 2, 3, and 5 bleeding and types 3 and 5 bleeding.

**Results:**

Of 15,013 patients, 4,606 (30.7%) were qualified as HBR and 8,395 (55.9%) had hsCRP >2 mg/L. Elevated hsCRP was consistently associated with higher risk of ischemic events in both HBR (adjusted hazard ratio [aHR]: 1.20; 95% confidence interval [CI]: 0.91–1.58) and non-HBR (aHR: 1.34; 95% CI: 1.01–1.78) subgroups (P _interaction_ = 0.755). Although the incidence of bleeding events was higher in HBR patients, an elevated hsCRP level was not associated with bleeding events regardless of HBR status. Restricted cubic spline regression represented an inverse J-shaped relation between hsCRP and non-HBR for ischemic events (P _nonlinearity_ <0.001) and all-cause death (P _nonlinearity_ = 0.003).

**Conclusion:**

Regardless of HBR status, high levels of hsCRP were associated with an increased risk of ischemic events and all-cause death in ACS patients following PCI, but not for bleeding.

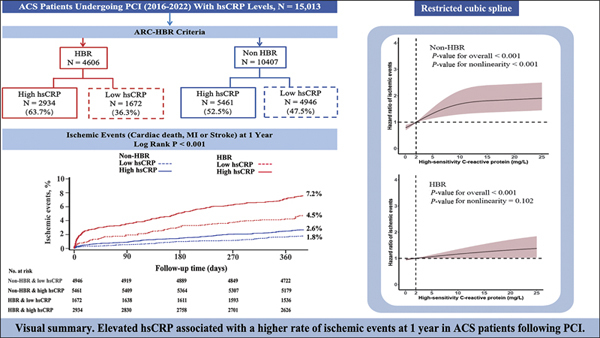

## Introduction

Even after undergoing a series of evidence-based therapeutic regimens, acute coronary syndrome (ACS) patients who received percutaneous coronary intervention (PCI) still carry significant residual risks of cardiovascular and thrombotic complications. 1 2 3 Consequently, it is extremely important to identify the potential residual risks and provide individualized management to enhance the prognosis of patients. As a biological pathway associated with residual thrombotic risk, systemic and vascular inflammation has garnered much more attention, given that recent studies have illustrated the beneficial effects of anti-inflammatory therapy in patients with atherosclerotic diseases. 4 5 6 7 8 9 Inflammation represents a crucial non-traditional risk factor for ACS, and further attention needs to be paid to how to deal with the residual inflammation risk. 10

Notably, the elevated level of inflammatory biomarkers can serve not only as a strong predictor of ischemic events but also as an indicator associated with a higher risk of bleeding in ACS patients undergoing PCI. 11 12 In this regard, several features of high bleeding risk (HBR) as defined by Academic Research Consortium (ARC), 13 such as renal insufficiency, cancer, advanced age, and anemia, have been proven to increase the inflammatory burden. 14 15 16 Given that ACS patients are routinely prescribed dual antiplatelet therapy (DAPT) following PCI, achieving a balance between the risks of ischemia and bleeding is of the utmost significance. 17 18 Nevertheless, it remains unclear whether there is a synergistic effect of bleeding and inflammation risks on both ischemic and bleeding events among patients treated with DAPT. Hence, we analyzed a large-sample, real-world PCI cohort to evaluate the potential influence of long-term outcomes according to the existence of bleeding and inflammation risk conditions in ACS patients who are receiving DAPT after PCI.

## Method

### Study Population and Design

The study cohort was derived from a prospective, real-world, single-center registry at the General Hospital of Northern Theater Command, which recruited consecutive patients who underwent PCI between March 2016 and March 2022. 17 The inclusion criteria were ACS patients who received DAPT following PCI, survived at discharge, and had available hsCRP levels. The exclusion criteria for this study were as follows 1 : patients with systemic inflammatory disease (such as systemic lupus erythematosus, sepsis, and severe infections), and 2 HBR criteria could not be determined owing to incomplete data. This study was approved by the institutional ethical committee of the General Hospital of Northern Theater Command and a waiver of the requirement to obtain informed consent was provided to conduct this analysis. The study also complied with the provisions of the Declaration of Helsinki.

### Laboratory Analysis and Data Collection

Blood samples of each patient were collected at admission, and all indicators were performed with standard hospital assays. The level of hsCRP was determined using a Cobas c 501 analyzer (Roche Diagnostics, Mannheim, Germany). 10 High inflammation risk was defined as hsCRP >2 mg/L. 19 Patients were stratified by the ARC-HBR criteria, which include a list of 20 clinical criteria—categorized into major and minor criteria—used to identify HBR in patients undergoing PCI. 9 13

### Outcomes and Follow-up

The primary outcome was ischemic events at 12 months, defined as a composite of cardiac death, myocardial infarction (MI), and/or stroke. The secondary outcomes include 12-month all-cause death, components of the ischemic event, Bleeding Academic Research Consortium (BARC) types 2, 3, and 5 bleeding, and types 3 and 5 bleeding. Clinical follow-ups were routinely conducted at 3, 6, 9, and 12 months after the procedure via phones, outpatient visits, or at unscheduled readmission by research staffs. Every clinical incident was reviewed by a clinical events committee.

### Statistical Analysis

Continuous variables were reported as the mean ± standard deviation (SD) or median (interquartile range) as appropriate and were compared using *t* -tests or the Wilcoxon rank sum test if nonnormally distributed. The Kolmogorov-Smirnov test and Q-Q plot were used to evaluate whether the data followed a normal distribution. Categorical variables were presented as numbers (percentages) and were compared using the χ2 test or Fisher's exact test. Time-to-event outcomes were analyzed by the Kaplan-Meier method and compared by the log-rank test. Cox proportional hazards models were used to estimate the hazard ratio (HR) and 95% confidence interval (CI) for each outcome among patients with or without HBR with comparisons performed between inflammation statuses. To address potential confounding factors, the following variables were selected for multivariate regression: age, sex, hypertension, diabetes, previous MI, previous stroke, previous PCI, peripheral artery disease, smoking status, presentation, estimated glomerular filtration rate (eGFR), anemia, and medical treatment at discharge.

The restricted cubic splines (RCS) were performed to explore the nonlinearity between hsCRP and 12-month ischemic events, all-cause death, and BARC types 2, 3, and 5 bleeding in HBR and non-HBR patients, respectively. Herein, UpSet plots were performed to illustrate the most frequent combinations of HBR major and minor criteria. The subgroup analyses were further stratified by the components of the HBR conditions to evaluate the impact of hsCRP. Unless otherwise noted, a two-sided *P* -value less than 0.05 indicated statistical significance. The statistical analysis was conducted using SAS software version 9.4 (SAS Institute, Cary, NC, USA) and R software version 4.2.0 ( http://www.r-project.org , Austria).

Sensitivity analyses included repeat analysis by using the PRECISE-DAPT score to evaluate bleeding risk, 20 and competing-risk analysis through Fine-Gray model accounting for the competitive risk of death and employing alternative hsCRP thresholds (1 and 3 mg/L).

## Results

### Study Population

**Fig. 1:**
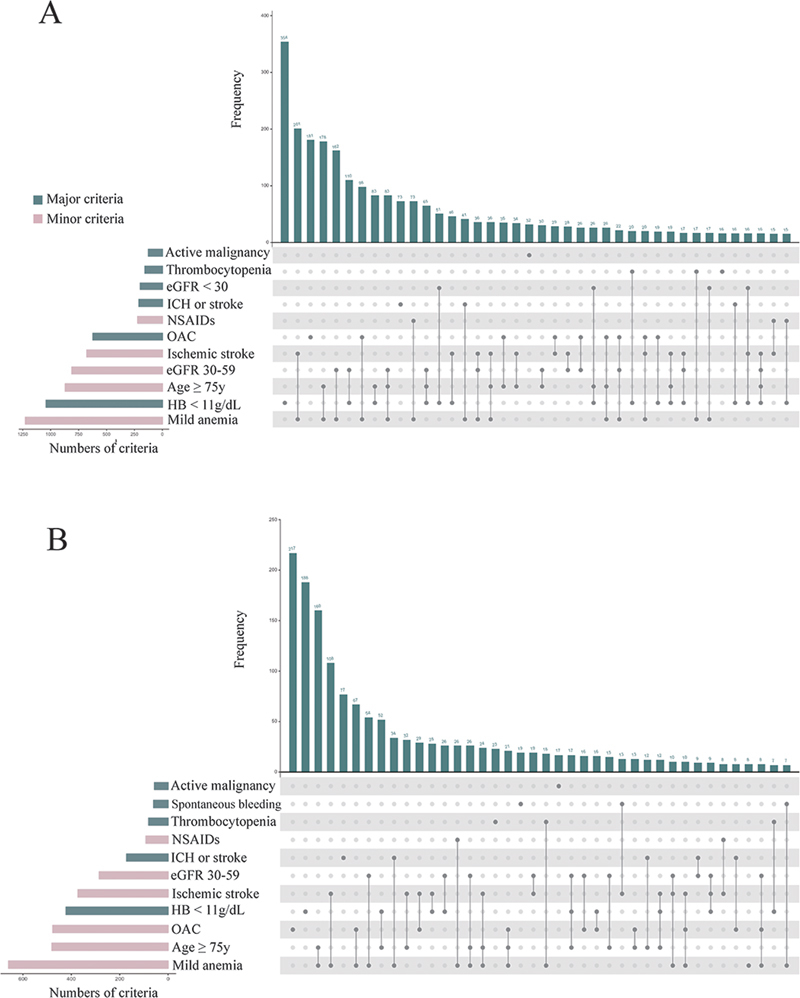
The UpSet plot shows the intersection of major and minor criteria of high bleeding risk HBR (e.g., mild anemia, oral anticoagulant [OAC], nonsteroidal anti-inflammatory drugs [NSAIDs]) in the study cohort. The bars on the bottom-left represent the frequency of each criterion (green–gray for major criteria and pink for minor criteria), and the connected dots indicate combinations of criteria. ( **A** ) High high-sensitivity C-reactive protein (hsCRP) group. ( **B** ) Low hsCRP group. eGFR, estimated glomerular filtration rate; ICH, intracerebral hemorrhage.

After screening 19,366 consecutive patients meeting the inclusion criteria, 15,013 patients enrolled in final analysis with a 99.3% follow-up rate at 12 months ( Supplementary Fig. S1 , available in the online version only). Among them, 4,606 patients (30.7%) met the ARC-HBR criteria. Moderate or severe anemia, taking oral anticoagulant agents, and prior moderate or severe cerebrovascular accident (CVA) were the first three major ARC-HBR criteria. The most common major ARC-HBR criterion was moderate or severe anemia (4.1 and 22.6% in the low and high hsCRP groups, respectively). Mild anemia, age ≥75 years old, and ischemic stroke were the first three minor ARC-HBR criteria. The most common minor ARC-HBR criterion was mild anemia (6.3 and 26.6% in the low and high hsCRP groups, respectively). The prevalence of each major and minor criteria and their most frequent combinations according to high and low hsCRP levels are presented in Fig. 1A and Fig. 1B , respectively.

Table 1 displays the baseline clinical and procedural characteristics stratified by HBR status and hsCRP levels. Among the 4,606 patients with HBR, 2,934 patients (63.7%) had elevated hsCRP levels, while it was elevated in 5,461 patients (52.5%) in the non-HBR group ( *p* < 0.0001). In both the non-HBR and HBR groups, patients with elevated hsCRP levels were more likely to be tobacco smokers, have acute MI at presentation, and less likely to have a history of MI and PCI. These patients also had lower ejection fraction, estimated glomerular filtration rate, and hemoglobin levels. The procedural information between the two groups was similar, with only numerical differences in the characteristics of stent implantations.

**Table 1:** Baseline characteristics of patients stratified by HBR and hsCRP

	Non-HBR ( *N* = 10,407)			HBR ( *N* = 4,606)		
	Low hsCRP (≤2 mg/L) ( *N* = 4,946)	High hsCRP (>2 mg/L) ( *N* = 5,461)	*P* -value	Low hsCRP (≤2 mg/L) ( *N* = 1,672)	High hsCRP (>2 mg/L) ( *N* = 2,934)	*P* -value
Age, years	58.7 ± 9.1	58.0 ± 10.4	<0.001	67.0 ± 10.3	67.3 ± 10.8	0.481
Male, %	3,966 (80.2%)	4,302 (78.8%)	0.076	1,082 (64.7%)	1,815 (61.9%)	0.054
Medical history, %						
Hypertension	2,666 (53.9%)	3,201 (58.7%)	<0.001	1,123 (67.2%)	2,020 (68.9%)	0.234
Diabetes	1,403 (28.4%)	1,680 (30.9%)	0.006	573 (34.4%)	1,078 (36.8%)	0.103
Previous MI	793 (16.1%)	671 (12.3%)	<0.001	344 (20.6%)	440 (15.1%)	<0.001
Previous stroke	262 (5.3%)	367 (6.7%)	0.002	542 (32.4%)	886 (30.2%)	0.121
Previous PCI	1,349 (27.3%)	872 (16.0%)	<0.001	545 (32.6%)	611 (20.9%)	<0.001
PAD	34 (0.7%)	39 (0.7%)	0.866	38 (2.3%)	60 (2.0%)	0.609
Smoking						
Never	2,022 (41.0%)	1,933 (35.5%)	<0.001	902 (54.1%)	1,397 (47.8%)	<0.001
Active	2,188 (44.4%)	2,950 (54.2%)		506 (30.4%)	1,123 (38.4%)	
Former	722 (14.6%)	556 (10.2%)		259 (15.5%)	402 (13.8%)	
Presentation, %						
UA	3,105 (62.8%)	2,053 (37.6%)	<0.001	1,025 (61.3%)	1,033 (35.2%)	<0.001
NSTEMI	549 (11.1%)	1,295 (23.7%)		209 (12.5%)	690 (23.5%)	
STEMI	1,292 (26.1%)	2,113 (38.7%)		438 (26.2%)	1,211 (41.3%)	
eGFR, mL/min/1.73 m ^2^	96.9 ± 20.7	95.4 ± 22.0	<0.001	83.0 ± 26.1	73.6 ± 29.1	<0.001
Anemia, %	1,126 (22.8%)	1,484 (27.2%)	<0.001	1,080 (64.6%)	2,264 (77.2%)	<0.001
hsCRP, mg/L	0.9 (0.5–1.4)	5.6 (3.3–12.2)	<0.001	1.0 (0.5–1.4)	7.8 (3.7–23.8)	<0.001
LVEF, %	58.4 ± 7.9	55.7 ± 9.0	<0.001	56.6 ± 9.0	53.2 ± 9.9	<0.001
Procedure information, %						
Transradial access	4,683 (94.7%)	5,142 (94.2%)	0.245	1,513 (90.5%)	2,592 (88.3%)	0.024
Coronary arteries treated						
LM	198 (4.0%)	187 (3.4%)	0.124	100 (6.0%)	156 (5.3%)	0.347
LAD	2,724 (55.1%)	2,868 (52.7%)	0.013	841 (50.4%)	1,393 (47.7%)	0.069
LCX	1,131 (22.9%)	1,336 (24.5%)	0.047	399 (23.9%)	674 (23.1%)	0.500
RCA	1,772 (35.9%)	2,009 (36.9%)	0.265	665 (39.9%)	1,278 (43.7%)	0.012
Number of stents	1.5 ± 0.9	1.5 ± 0.9	0.005	1.5 ± 1.0	1.5 ± 1.0	0.025
Total length of stents, mm	41.8 ± 24.3	42.9 ± 24.7	0.007	43.4 ± 25.6	45.2 ± 25.5	0.008
Average stent diameters, mm	3.0 ± 0.4	3.0 ± 0.4	<0.001	3.0 ± 0.4	3.0 ± 0.4	0.977
Medications at discharge, %						
Aspirin	4,833 (97.7%)	5,272 (96.5%)	<0.001	1,497 (89.5%)	2,668 (90.9%)	0.120
P2Y12 inhibitors						
Clopidogrel	3,125 (63.2%)	3,308 (60.6%)	0.006	1,330 (79.5%)	2,296 (78.3%)	0.303
Ticagrelor	1,821 (36.8%)	2,153 (39.4%)		342 (20.5%)	638 (21.7%)	
Statins	4,502 (91.0%)	4,830 (88.4%)	<0.001	1,499 (89.7%)	2,564 (87.4%)	0.022
ACEI/ARB	2,591 (52.4%)	3,280 (60.1%)	<0.001	905 (54.1%)	1,581 (53.9%)	0.875
Β-blockers	2,955 (59.7%)	3,540 (64.8%)	<0.001	1,013 (60.6%)	1,797 (61.2%)	0.658
PPI	1,600 (32.3%)	1,801 (33.0%)	0.494	647 (38.7%)	1,054 (35.9%)	0.061
OAC	0 (0.0%)	0 (0.0%)	–	476 (28.5%)	622 (21.2%)	<0.001

Abbreviations: ACEI, angiotensin-converting enzyme inhibitors; ARB, angiotensin II receptor blocker; eGFR, estimated glomerular filtration rate; HBR, high bleeding risk; hsCRP, high-sensitivity C-reaction protein; LAD, left anterior descending; LCX, left circumflex; LM, left main; LVEF, left ventricular ejection fraction; MI, myocardial infarction; NSTEMI, non-ST-elevation myocardial infarction; OAC, oral anticoagulants; PAD, peripheral vascular disease; PCI, percutaneous coronary intervention; PPI, proton pump inhibitors; RCA, right coronary artery; STEMI, ST-elevation myocardial infarction; UA, unstable angina.

### Clinical Outcomes According to hsCRP and ARC-HBR Criteria

**Fig. 2:**
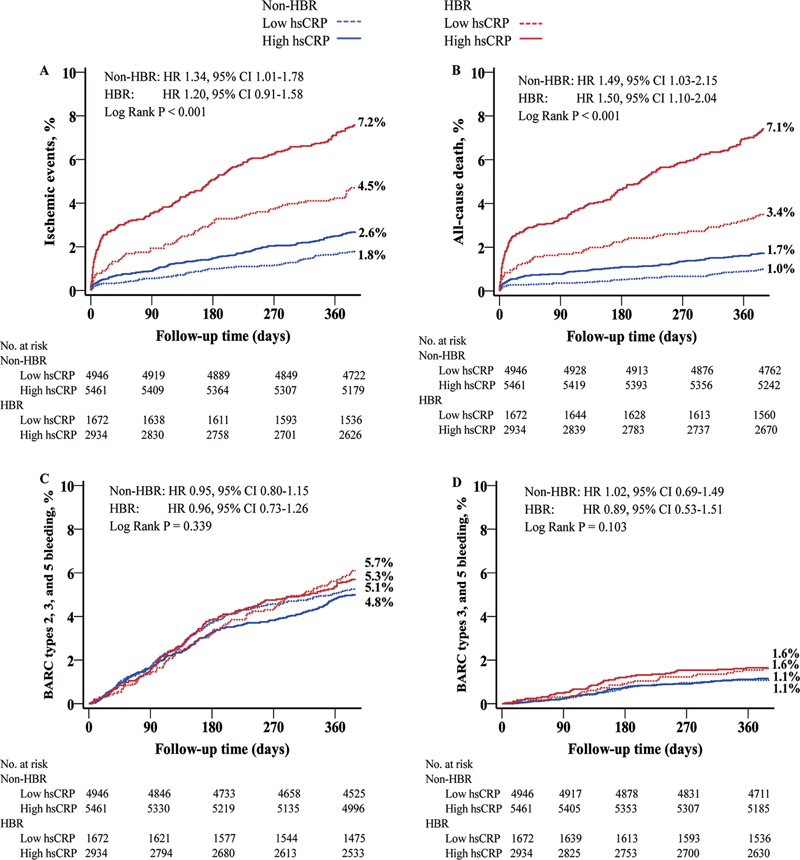
Kaplan–Meier curves for the primary and secondary outcomes across bleeding and inflammatory burden. ( **A** ) Ischemic events; ( **B** ) all-cause death; ( **C** ) Bleeding Academic Research Consortium (BARC) types 2, 3, and 5 bleeding; and ( **D** ) BARC types 3 and 5 bleeding. HBR, high bleeding risk; hsCRP, high-sensitivity C-reactive protein.

The primary outcome of ischemic events at 1 year was significantly higher in patients with high hsCRP and HBR, compared to patients with low hsCRP and non-HBR (low vs. high hsCRP: non-HBR, 1.8% vs. 2.6%; HBR, 4.5% vs. 7.2%; *p* < 0.001) ( Fig. 2 ). Patients with high hsCRP had a relatively higher risk of ischemic events regardless of HBR status (non-HBR: adjusted hazard ratio [aHR] 1.34, 95% confidence interval [CI] 1.01–1.78, *p* = 0.041; HBR: aHR 1.20, 95% CI 0.91–1.58, *p* = 0.201; P for interaction = 0.755) ( Table 2 ). Regarding the all-cause death outcome, high hsCRP was associated with an increased risk compared with low hsCRP (non-HBR: aHR 1.49, 95% CI 1.03–2.15, *p* = 0.033; HBR: aHR 1.50, 95% CI 1.10–2.04, *p* = 0.010). No significant differences in bleeding outcomes were observed among the four groups. Moreover, no interaction effects were identified between HBR status and hsCRP levels for either primary or secondary outcomes (all P _interaction_ > 0.05). The adjusted association between HBR and adverse events at 1 year, stratified by hsCRP levels, is summarized in Supplementary Table S1 (available in the online version only). Sensitivity analyses using alternative cut-off values for hsCRP (1 and 3 mg/L) 21 22 yielded results consistent with the primary analysis, reaffirming the association between elevated hsCRP and increased ischemic risk ( Supplementary Table S2 , available in the online version only). Consistent results were observed from competing risk model and Cox proportional hazard model ( Supplementary Table S3 , available in the online version only). An elevated PRECISE-DAPT score was significantly associated with an increased risk of BARC types 3 and 5 bleeding (1.9% vs. 1.2%, HR: 1.71, 95%CI, 1.19–2.45, *p* = 0.004) ( Supplementary Table S4 , available in the online version only). Besides following the adjustment for baseline covariates, higher levels of hsCRP were associated with increased incidences of ischemic events and all-cause death stratified by PRECISE-DAPT score ( Supplementary Table S5 , available in the online version only).

**Table 2:** Outcomes of individuals stratified by HBR and hsCRP at 1 year

	Non-HBR ( *N* = 10,407)			*P* -value	HBR ( *N* = 4,606)				P for interaction
	Low hsCRP (≤2 mg/L) ( *N* = 4,946)	High hsCRP (>2 mg/L) ( *N* = 5,461)	Adjusted HR (95%CI)		Low hsCRP (≤2 mg/L) ( *N* = 1,672)	High hsCRP (>2 mg/L) ( *N* = 2,934)	Adjusted HR (95%CI)	*P* -value	
Ischemic events	87 (1.8%)	144 (2.6%)	1.34 (1.01–1.78)	0.041	76 (4.5%)	212 (7.2%)	1.20 (0.91–1.58)	0.201	0.755
Cardiac death	44 (0.9%)	77 (1.4%)	1.29 (0.87–1.92)	0.200	46 (2.8%)	162 (5.5%)	1.34 (0.95–1.89)	0.097	0.843
MI	23 (0.5%)	36 (0.7%)	1.42 (0.82–2.47)	0.214	8 (0.5%)	24 (0.8%)	1.35 (0.57–3.20)	0.492	0.794
Stroke	24 (0.5%)	38 (0.7%)	1.45 (0.84–2.50)	0.180	25 (1.5%)	30 (1.0%)	0.80 (0.46–1.40)	0.432	0.053
All-cause death	49 (1.0%)	94 (1.7%)	1.49 (1.03–2.15)	0.033	57 (3.4%)	208 (7.1%)	1.50 (1.10–2.04)	0.010	0.942
BARC types 2, 3, and 5 bleeding	252 (5.1%)	262 (4.8%)	0.95 (0.80–1.15)	0.615	96 (5.7%)	155 (5.3%)	0.96 (0.73–1.26)	0.758	0.962
BARC types 3 and 5 bleeding	54 (1.1%)	62 (1.1%)	1.02 (0.69–1.49)	0.939	26 (1.6%)	46 (1.6%)	0.89 (0.53–1.51)	0.673	0.848

Abbreviations: BARC, Bleeding Academic Research Consortium; CI, confidence interval; eGFR, estimated glomerular filtration rate; HBR, high bleeding risk; HR, hazard ratio; hsCRP, high-sensitivity C-reactive protein; MI, myocardial infarction; PAD, peripheral arterial disease; PCI, percutaneous coronary intervention.

Note: Model adjusted for age, sex, hypertension, diabetes, previous MI, previous stroke, previous PCI, PAD, smoking status, presentation, eGFR, anemia, and medical treatment at discharge.

Forest plots provided the primary outcome of ischemic events, all-cause death, and BARC types 2, 3, and 5 bleeding at 12 months based on major and minor criteria for ARC-HBR and prehospital statin use ( Supplementary Figs. S2 S3 S4 , available in the online version only). No statistically significant interactions were detected between all the major and minor criteria of HBR and different levels of hsCRP (all P _interaction_ > 0.05) for BARC types 2, 3, and 5 bleeding. However, a significant difference was found in the major criteria of taking oral anticoagulant (OAC) agents (ischemic events: OAC: HR 2.94, 95% CI 1.63–5.31; No OAC: HR 1.35, 95% CI 1.01–1.81, P _interaction_ = 0.02; all-cause death: OAC: HR 5.99, 95% CI 2.73–13.18; No OAC: HR 1.62, 95% CI 1.18–2.23, P _interaction_ < 0.01), which demonstrated an interaction between high bleeding status and high inflammatory burden for ischemic events and all-cause death ( Supplementary Figs. S2 and S3 , available in the online version only).

### Nonlinear Association between hsCRP and Outcomes

**Fig. 3:**
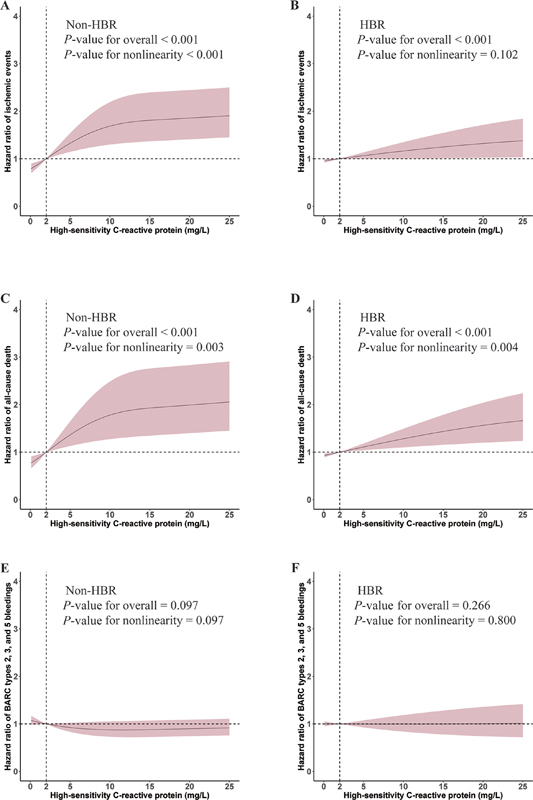
Restricted cubic spline for the association between high-sensitivity C-reactive protein (hsCRP) and ischemic events ([ **A** ] non–high bleeding risk [non-HBR] patients and [ **B** ] HBR patients), all-cause death ([ **C** ] non-HBR patients and [ **D** ] HBR patients), and Bleeding Academic Research Consortium (BARC) types 2, 3, and 5 bleeding ([ **E** ] non-HBR patients and [ **F** ] HBR patients).

Patients were stratified into four groups based on the hsCRP level and HBR status. Using RCS ( Fig. 3 ), we identified an inverse J-shaped relationship between hsCRP and non-HBR in ischemic events (P for non-linearity <0.001) and all-cause death (P for non-linearity = 0.003). In non-HBR group, the incidence of both ischemic events and all-cause death rises significantly at the outset and then gradually rises with the increased level of hsCRP (both *p* < 0.001). In the HBR patients, the risk of ischemic events ( *p* < 0.001) and all-cause death ( *p* < 0.001) manifested a slow upward trend with the increase of hsCRP, but a significant difference was still seen at the cut-off of 2 mg/L. RCS analysis suggests a possible linear relationship between hsCRP and BARC types 2, 3, and 5 bleeding events in both HBR (P for nonlinearity = 0.800) and non-HBR (P for nonlinearity = 0.097).

## Discussion

This study, leveraging data from a large real-world registry, examined the integration of hsCRP and HBR criteria to enhance risk stratification in ACS patients undergoing PCI with contemporary therapies. Key findings include 1 : The impact of hsCRP on different HBR criteria and the composition of the HBR population varies. 2 Non-HBR showed a nonlinear relationship with hsCRP in ischemic events while HBR did not. 3 Among the people at high risk of bleeding, patients with high hsCRP account for a high proportion (63.7%), suggesting a promise in future for anti-inflammatory therapy. 4 HBR and high hsCRP group was associated with a fourfold increase in risk of ischemic events in ACS after PCI compared with non-HBR and low hsCRP group, without significant difference in bleeding events.

In this study, the HBR component of the ACS population revealed that oral anticoagulants and anemia were the most prevalent major criteria, and anemia and age ≥75 years were the most common minor criteria. It can be explained by the heterogeneity of patient profiles among different studies. Compared with previous studies, patients in the present study were younger and had fewer comorbidities (e.g., malignancy, thrombocytopenia), resulting in a lower overall incidence of HBR. 11 23 24 25 Consequently, advanced age, improved adherence to chronic OAC treatment, and the presence of anemia have emerged as the principal factors contributing to HBR within this patient cohort. Furthermore, the heightened focus on OAC treatment within clinical guidelines, coupled with a marked increase in patient awareness and adherence to such therapies in China, substantially contributed to the larger proportion of OAC use as the cause of HBR. 26 Moreover, previous study showed persistent systemic inflammation was associated with major bleeding risk in atrial fibrillation patients. 27

RCS plots revealed a nonlinear relationship between hsCRP and ischemic events in non-HBR patients but a linear relationship in the HBR population. Conditions such as anemia, chronic kidney disease, and active malignancy in past 12 months, which are associated with systemic inflammation, were prevalent among HBR patients and correlated with worse outcomes as hsCRP levels increased. 28 29 30 A notable finding in the present study was that the inflammation did not impact the risk of bleeding events, regardless of HBR status. This finding can offer valuable insights into current clinical practice for administering anti-inflammatory agents to reduce the risk of ischemic events and all-cause death in ACS patients underwent PCI without too much consideration to bleeding complications. Nevertheless, the correlation between hsCRP and adverse events tended to be stable in the non-HBR population, and the incremental factors associated with hsCRP were significantly less than those in the HBR population.

Inflammation plays an essential role in the formation of atherosclerotic plaques in the coronary arteries and the incomplete stent apposition post PCI. 31 32 Recent meta-analysis shows that in secondary prevention for MI, anti-inflammatory therapy significantly reduces Major Adverse Cardiovascular Events (MACE) without increasing serious adverse events. 33 Recent prospective study of colchicine found favorable effects on coronary plaque stabilization at optical coherence tomography in ACS patients. 34 In addition, several clinical studies have shown that the hsCRP level representing residual inflammatory burden in East Asian patients with ACS is lower than that in the Western population, suggesting the need for population-specific hsCRP cutoffs to refine prognostic models. 19 21 35 36 To reduce the risk of thrombosis in HBR population, antiplatelet therapy may be a concern. However, anti-inflammatory therapy can reduce thrombotic events without increasing the risk of bleeding. Genetic differences, such as CYP2C19 polymorphisms common in East Asians, predispose this population to higher bleeding risks and lower ischemic risks, further emphasizing the need for personalized therapies. 37

Although anti-inflammatory therapy has been repeatedly proposed for secondary prevention in patients with ACS, studies have not clarified in patients with ACS at high risk of bleeding. 38 39 Previous cohort of Mount Sinai Hospital, a the large, single-center prospective study, which showed that hsCRP was associated with higher MACE events but not associated with bleeding risk, regardless of HBR status. 11 In our study, the incidence of ischemic events and all-cause mortality was significantly higher in HBR patients than in the non-HBR population, and higher in the population with high risk of inflammation, among which the main components of ischemic events were acceptable. In the cardiac death event, followed by stroke, this may be related to the high proportion of clopidogrel in China. A retrospective analysis of a multicenter prospective cohort study showed that systemic inflammation in the acute phase of MI was an independent risk factor for cardiovascular events, but not a related cause of bleeding. 14

Large meta-analyses have shown that anti-inflammatory therapy for acute MI and stable coronary artery disease can reduce the risk of stroke, and anti-inflammatory therapy for stroke patients has also been studied. 40 Whether cardiovascular anti-inflammatory therapy can have benefits in cerebrovascular diseases is also worth exploring. Our study found that the overall inflammation level in patients with HBR was higher than that in ordinary patients with ACS, and there was an interaction between all-cause death and previous cerebrovascular events, suggesting that inflammation may play a bridging role. Therefore, the focus of future anti-inflammatory therapies in patients with ACS who are at HBR can also be beneficial, and more prospective studies are needed to explore the specific anti-inflammatory therapy options and regimens in this population.

HBR people also have ischemic risk, so clinicians need to consider more about the patients' benefit and bleeding risk when making antithrombotic decisions. The OPT-BIRISK Trial provided a basis for treatment strategies for patients at high risk of both ischemia and hemorrhage. 41 Our study is based on an East Asian population with characteristics such as hypocoagulability and low inflammation, which may be one of the key factors for its low risk of ischemia, while factors such as the high conversion rate of *Helicobacter pylori* infection, intracranial atherosclerosis, and post-stroke hemorrhage may be associated with a high risk of gastrointestinal bleeding and intracranial hemorrhage during antithrombotic therapy. The preference for conservative P2Y12 inhibitors in this population may explain the low bleeding event rates observed in our cohort. 42 Nonetheless, our findings are consistent with previous studies in cardiovascular disease populations, reinforcing the importance of personalized treatment strategies in these patients.

## Limitations

Our study has several limitations. First, our study is a post hoc analysis of a sizable prospective single-center cohort of ACS patients who underwent PCI, which may impact the generalizability of the results. These findings require confirmation through more specifically designed studies. However, the results of our study remain clinically and biologically plausible and align with those obtained from the United States. 11 Second, the rate of major bleeding events was lower than expected according to the ARC-HBR criteria at 4% of BARC types 3 or 5 bleeding at 12 month. 13 Third, circulating hsCRP levels were measured at the time of hospitalization, which may not reflect average levels during follow-up. On-treatment data may hold greater clinical significance. Furthermore, this study did not implement targeted anti-inflammatory treatments for patients with high levels of inflammation.

## Conclusion

In summary, the current study indicated that, irrespective of HBR status, elevated levels of hsCRP were associated with an augmented risk of ischemic events, and all-cause mortality, but not for bleeding events in ACS patients receiving DAPT following PCI. Thus, anti-inflammatory therapies could have potential benefits, offering the reduction of thrombotic events without appreciable effect on bleeding in these patients.

## Notes

**What is known about this topic?**

- The beneficial effects of anti-inflammatory therapy in ACS patients after PCI include reduction in residual thrombotic risk.
- Conditions in the ARC-HBR criteria are related to proinflammatory status.

**What does this paper add?**

- Elevated hsCRP level is associated with a higher rate of ischemic events but not bleeding events at 1 year.
- There is no interaction between HBR status and different hsCRP levels.

## References

[1] Bay B Vogel B Sharma R Inflammatory risk and clinical outcomes according to polyvascular atherosclerotic disease status in patients undergoing PCI Clin Res Cardiol 2025 114 08 969 977 38900274 10.1007/s00392-024-02471-w

[2] Mahmoud A K Farina J M Awad K Lipoprotein(a) and long-term in-stent restenosis after percutaneous coronary intervention Eur J Prev Cardiol 2024 31 15 1878 1887 38916491 10.1093/eurjpc/zwae212

[3] Alkhalil M Kuzemczak M Zhao R Prognostic role of residual thrombus burden following thrombectomy: insights from the TOTAL trial Circ Cardiovasc Interv 2022 15 05 e011336 35580203 10.1161/CIRCINTERVENTIONS.121.011336

[4] ESC Scientific Document Group Byrne R A Rossello X Coughlan J J 2023 ESC Guidelines for the management of acute coronary syndromes Eur Heart J Acute Cardiovasc Care 2024 13 01 55 161 37740496 10.1093/ehjacc/zuad107

[5] CANTOS Trial Group Ridker P M Everett B M Thuren T Antiinflammatory therapy with canakinumab for atherosclerotic disease N Engl J Med 2017 377 12 1119 1131 28845751 10.1056/NEJMoa1707914

[6] Tardif J C Kouz S Waters D D Efficacy and safety of low-dose colchicine after myocardial infarction N Engl J Med 2019 381 26 2497 2505 31733140 10.1056/NEJMoa1912388

[7] LoDoCo2 Trial Investigators Nidorf S M Fiolet A TL Mosterd A Colchicine in patients with chronic coronary disease N Engl J Med 2020 383 19 1838 1847 32865380 10.1056/NEJMoa2021372

[8] Bouabdallaoui N Tardif J C Waters D D Time-to-treatment initiation of colchicine and cardiovascular outcomes after myocardial infarction in the Colchicine Cardiovascular Outcomes Trial (COLCOT) Eur Heart J 2020 41 42 4092 4099 32860034 10.1093/eurheartj/ehaa659 PMC7700755

[9] Ridker P M MacFadyen J G Glynn R J Bradwin G Hasan A A Rifai N Comparison of interleukin-6, C-reactive protein, and low-density lipoprotein cholesterol as biomarkers of residual risk in contemporary practice: secondary analyses from the Cardiovascular Inflammation Reduction Trial Eur Heart J 2020 41 31 2952 2961 32221587 10.1093/eurheartj/ehaa160 PMC7453833

[10] Liao J Qiu M Su X The residual risk of inflammation and remnant cholesterol in acute coronary syndrome patients on statin treatment undergoing percutaneous coronary intervention Lipids Health Dis 2024 23 01 172 38849939 10.1186/s12944-024-02156-3 PMC11157837

[11] Vinayak M Cao D Tanner R Impact of bleeding risk and inflammation on cardiovascular outcomes after percutaneous coronary intervention JACC Cardiovasc Interv 2024 17 03 345 355 38355263 10.1016/j.jcin.2023.12.004

[12] Chen R Liu C Zhou P Both low and high postprocedural hsCRP associate with increased risk of death in acute coronary syndrome patients treated by percutaneous coronary intervention Mediators Inflamm 2020 2020 9.343475E6 10.1155/2020/9343475 PMC7183527 32377168

[13] Urban P Mehran R Colleran R Defining high bleeding risk in patients undergoing percutaneous coronary intervention: a consensus document from the Academic Research Consortium for High Bleeding Risk Circulation 2019 140 03 240 261 31116395 10.1093/eurheartj/ehz372 PMC6736433

[14] Nanchen D Klingenberg R Gencer B Inflammation during acute coronary syndromes—risk of cardiovascular events and bleeding Int J Cardiol 2019 287 13 18 31003794 10.1016/j.ijcard.2019.03.049

[15] Conference Participants Sarnak M J Amann K Bangalore S Chronic kidney disease and coronary artery disease: JACC state-of-the-art review J Am Coll Cardiol 2019 74 14 1823 1838 31582143 10.1016/j.jacc.2019.08.1017

[16] Zheng Y Huang Y Li H Hemoglobin albumin lymphocyte and platelet score and all-cause mortality in coronary heart disease: a retrospective cohort study of NHANES database Front Cardiovasc Med 2023 10 1.241217E6 10.3389/fcvm.2023.1241217 PMC10679332 38028472

[17] Qiu M Na K Qi Z Contemporary use of ticagrelor vs clopidogrel in patients with acute coronary syndrome undergoing percutaneous coronary intervention: a GRACE risk score stratification-based analysis in a large-scale, real-world study from China Mayo Clin Proc 2023 98 07 1021 1032 37419570 10.1016/j.mayocp.2023.02.004

[18] Na K Qiu M Ma S Impact of ticagrelor vs. clopidogrel in patients with acute coronary syndrome undergoing percutaneous coronary intervention after risk stratification with the CHA _2_ DS _2_ -VASc score Front Cardiovasc Med 2022 9 808571 35445091 10.3389/fcvm.2022.808571 PMC9013766

[19] Ahn J-H Tantry U S Kang M G Residual inflammatory risk and its association with events in East Asian patients after coronary intervention JACC Asia 2022 2 03 323 337 36338415 10.1016/j.jacasi.2021.11.014 PMC9627808

[20] PRECISE-DAPT Study Investigators Costa F van Klaveren D James S Derivation and validation of the predicting bleeding complications in patients undergoing stent implantation and subsequent dual antiplatelet therapy (PRECISE-DAPT) score: a pooled analysis of individual-patient datasets from clinical trials Lancet 2017 389(10073):1025 1034 28290994 10.1016/S0140-6736(17)30397-5

[21] Yu M Yuan Y-F Yang F Residual inflammatory risk in outcomes of Chinese patients after percutaneous coronary intervention JACC Asia 2024 4 08 636 638 39156507 10.1016/j.jacasi.2024.05.004 PMC11328748

[22] Centers for Disease Control and Prevention American Heart Association Pearson T A Mensah G A Alexander R W Markers of inflammation and cardiovascular disease: application to clinical and public health practice: a statement for healthcare professionals from the Centers for Disease Control and Prevention and the American Heart Association Circulation 2003 107 03 499 511 12551878 10.1161/01.cir.0000052939.59093.45

[23] Montalto C Munafò A R Arzuffi L Validation of the ARC-HBR criteria in 68,874 patients undergoing PCI: a systematic review and meta-analysis Hellenic J Cardiol 2022 66 59 66 35550178 10.1016/j.hjc.2022.04.008

[24] Nakamura M Kadota K Nakao K High bleeding risk and clinical outcomes in East Asian patients undergoing percutaneous coronary intervention: the PENDULUM registry EuroIntervention 2021 16 14 1154 1162 32624464 10.4244/EIJ-D-20-00345 PMC9725071

[25] Gragnano F Spirito A Corpataux N Impact of clinical presentation on bleeding risk after percutaneous coronary intervention and implications for the ARC-HBR definition EuroIntervention 2021 17 11 e898 e909 34105513 10.4244/EIJ-D-21-00181 PMC9725019

[26] Du X Guo L Xia S Atrial fibrillation prevalence, awareness and management in a nationwide survey of adults in China Heart 2021 107 07 535 541 33509976 10.1136/heartjnl-2020-317915 PMC7958113

[27] Hamanaka Y Sotomi Y Hirata A Persistent systemic inflammation is associated with bleeding risk in atrial fibrillation patients Circ J 2020 84 03 411 418 32051386 10.1253/circj.CJ-19-1006

[28] Steinvil A Rogowski O Banai S Anemia and inflammation have an additive value in risk stratification of patients undergoing coronary interventions J Cardiovasc Med (Hagerstown) 2015 16 02 106 111 23846678 10.2459/JCM.0b013e32836380b4

[29] Ridker P M Tuttle K R Perkovic V Libby P MacFadyen J G Inflammation drives residual risk in chronic kidney disease: a CANTOS substudy Eur Heart J 2022 43 46 4832 4844 35943897 10.1093/eurheartj/ehac444

[30] Libby P Kobold S Inflammation: a common contributor to cancer, aging, and cardiovascular diseases-expanding the concept of cardio-oncology Cardiovasc Res 2019 115 05 824 829 30830168 10.1093/cvr/cvz058 PMC6452304

[31] Attizzani G F Capodanno D Ohno Y Tamburino C Mechanisms, pathophysiology, and clinical aspects of incomplete stent apposition J Am Coll Cardiol 2014 63 14 1355 1367 24530675 10.1016/j.jacc.2014.01.019

[32] Tucker B Vaidya K Cochran B J Patel S Inflammation during percutaneous coronary intervention-prognostic value, mechanisms and therapeutic targets Cells 2021 10 06 1391 34199975 10.3390/cells10061391 PMC8230292

[33] Laudani C Occhipinti G Greco A Giacoppo D Spagnolo M Capodanno D A pairwise and network meta-analysis of anti-inflammatory strategies after myocardial infarction: the TITIAN study Eur Heart J Cardiovasc Pharmacother 2025 pvae100 10.1093/ehjcvp/pvae100 PMC12046582 39756386

[34] Yu M Yang Y Dong S L Effect of Colchicine on Coronary Plaque Stability in Acute Coronary Syndrome as Assessed by Optical Coherence Tomography: The COLOCT Randomized Clinical Trial Circulation 2024 150 13 981 993 39166327 10.1161/CIRCULATIONAHA.124.069808

[35] Kawada-Watanabe E Yamaguchi J Sekiguchi H Arashi H Ogawa H Hagiwara N Targeting high-sensitivity C-reactive protein levels in acute coronary syndrome patients undergoing contemporary lipid-lowering therapy: a sub-analysis of the HIJ-PROPER trial J Cardiol 2020 75 05 500 506 31699568 10.1016/j.jjcc.2019.09.015

[36] Takahashi N Dohi T Endo H Residual inflammation indicated by high-sensitivity C-reactive protein predicts worse long-term clinical outcomes in Japanese patients after percutaneous coronary intervention J Clin Med 2020 9 04 1033 32268533 10.3390/jcm9041033 PMC7230848

[37] PTRG-DES consortium investigators Kim S E Jeon H S Go T H High platelet reactivity combined with CYP2C19 genotype in predicting outcomes in East Asian patients undergoing percutaneous coronary intervention Clin Pharmacol Ther 2023 114 05 1104 1115 37597219 10.1002/cpt.3026

[38] Feng J Wu Y Interleukin-35 ameliorates cardiovascular disease by suppressing inflammatory responses and regulating immune homeostasis Int Immunopharmacol 2022 110 108938 35759811 10.1016/j.intimp.2022.108938

[39] Wang H Liu Z Shao J Immune and inflammation in acute coronary syndrome: molecular mechanisms and therapeutic implications J Immunol Res 2020 2020 4.904217E6 10.1155/2020/4904217 PMC7450309 32908939

[40] Kelly P J Lemmens R Tsivgoulis G Inflammation and stroke risk: a new target for prevention Stroke 2021 52 08 2697 2706 34162215 10.1161/STROKEAHA.121.034388

[41] OPT-BIRISK Investigators Li Y Li J Wang B Extended clopidogrel monotherapy vs DAPT in patients with acute coronary syndromes at high ischemic and bleeding risk: the OPT-BIRISK randomized clinical trial JAMA Cardiol 2024 9 06 523 531 38630489 10.1001/jamacardio.2024.0534 PMC11024736

[42] Kim H K Tantry U S Smith S C Jr The East Asian paradox: an updated position statement on the challenges to the current antithrombotic strategy in patients with cardiovascular disease Thromb Haemost 2021 121 04 422 432 33171520 10.1055/s-0040-1718729

