# Supplementary material for: Inflammatory and Bleeding Risks on Clinical Outcomes in Acute Coronary Syndrome Patients Undergoing Percutaneous Coronary Intervention
Source: Thromb Haemost. 2025 Mar 13;125(12):1256–65. doi: 10.1055/a-2531-3268 (PMC12659991; doi:10.1055/a-2531-3268)
Supplement: Supplementary file 1 — Supplementary Material [file 10-1055-a-2531-3268-s24110618.pdf]

## Supplementary Materials

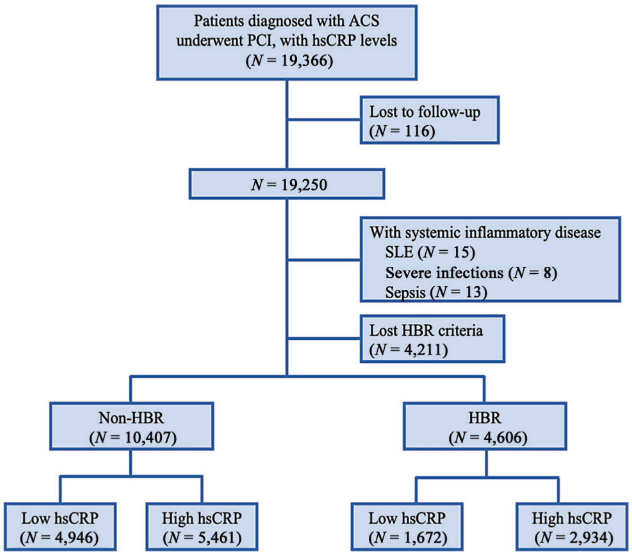


**Supplementary Fig. S1** Flowchart of this study.

 © 2025. The Author(s).

| | Ischemic events - *Events/Total (%)* hsCRP ≤2 mg/L | hsCRP >2 mg/L | Hazard ratio (95%CI) | P for interaction |
|---|---|---|---|---|
| Oral anticoagulation | | | | 0.02 |
| Yes | 14/476 (2.9) | 52/622 (8.4) | 2.94 (1.63-5.31) | |
| No | 62/1196 (5.2) | 160/2312 (6.9) | 1.35 (1.01-1.81) | |
| Severe or end-stage CKD | | | | 0.34 |
| Yes | 9/43 (20.9) | 44/199 (22.1) | 1.05 (0.51-2.14) | |
| No | 67/1629 (4.1) | 168/2735 (6.1) | 1.51 (1.14-2.01) | |
| Moderate or severe anemia | | | | 0.93 |
| Yes | 23/421 (5.5) | 89/1039 (8.6) | 1.60 (1.01-2.52) | |
| No | 53/1251 (4.2) | 123/1895 (6.5) | 1.56 (1.13-2.15) | |
| Spontaneous bleeding | | | | 0.53 |
| Yes | 4/59 (6.8) | 6/80 (7.5) | 1.09 (0.31-3.88) | |
| No | 72/1613 (4.5) | 206/2854 (7.2) | 1.65 (1.26-2.15) | |
| Moderate or severe thrombocytopenia | | | | 0.34 |
| Yes | 8/79 (10.1) | 17/154 (11.0) | 1.08 (0.46-2.49) | |
| No | 68/1593 (4.3) | 195/2780 (7.0) | 1.67 (1.27-2.21) | |
| Chronic bleeding diathesis | | | | 0.96 |
| Yes | 1/7 (14.3) | 0/6 (0.0) | - | |
| No | 75/1665 (4.5) | 212/2928 (7.2) | 1.64 (1.26-2.13) | |
| Active malignancy | | | | 0.76 |
| Yes | 2/55 (3.6) | 9/124 (7.3) | 2.04 (0.44-9.45) | |
| No | 74/1617 (4.6) | 203/2810 (7.2) | 1.61 (1.23-2.10) | |
| Previous cerebrovascular accident (major) | | | | 0.69 |
| Yes | 9/171 (5.3) | 15/211 (7.1) | 1.38 (0.60-3.15) | |
| No | 67/1501 (4.5) | 197/2723 (7.2) | 1.65 (1.25-2.17) | |
| Recent major surgery | | | | 0.97 |
| Yes | 0/12 (0.0) | 1/20 (5.0) | - | |
| No | 76/1660 (4.6) | 211/2914 (7.2) | 1.61 (1.24-2.09) | |
| Age ≥75 years | | | | 0.79 |
| Yes | 28/480 (5.8) | 83/868 (9.6) | 1.68 (1.10-2.58) | |
| No | 48/1192 (4.0) | 129/2066 (6.2) | 1.57 (1.13-2.19) | |
| Moderate CKD | | | | 0.57 |
| Yes | 20/284 (7.0) | 76/809 (9.4) | 1.35 (0.83-2.21) | |
| No | 56/1388 (4.0) | 136/2125 (6.4) | 1.61 (1.18-2.20) | |
| Mild anemia | | | | 0.91 |
| Yes | 29/659 (4.4) | 84/1225 (6.9) | 1.59 (1.04-2.42) | |
| No | 47/1013 (4.6) | 128/1709 (7.5) | 1.64 (1.17-2.29) | |
| Oral NSAIDs or steroids | | | | 0.75 |
| Yes | 4/90 (4.4) | 13/219 (5.9) | 1.35 (0.44-4.15) | |
| No | 72/1582 (4.6) | 199/2715 (7.3) | 1.64 (1.25-2.15) | |
| Previous cerebrovascular accident (minor) | | | | 0.69 |
| Yes | 18/371 (4.9) | 57/675 (8.4) | 1.77 (1.04-3.01) | |
| No | 58/1301 (4.5) | 155/2259 (6.9) | 1.57 (1.16-2.12) | |
| Prehospital statin use | | | | 0.75 |
| Yes | 23/560 (4.1) | 46/763 (6.0) | 1.49 (0.90-2.46) | |
| No | 53/1112 (4.8) | 166/2171 (7.6) | 1.63 (1.20-2.22) | |

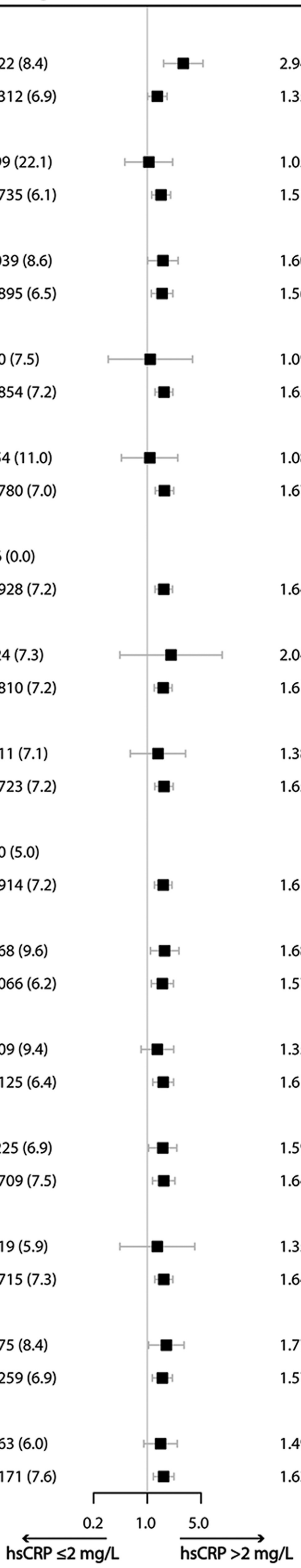


**Supplementary Fig. S2** Forest plot showing associations between high-sensitivity C-reactive protein (hsCRP) and ischemic events stratified by components of Academic Research Consortium–high bleeding risk (ARC-HBR) criteria and prehospital statin therapy.

 © 2025. The Author(s).

| | All-cause death - *Events/Total (%)* | | Hazard ratio (95%CI) | P for interaction |
|---|---|---|---|---|
| | hsCRP ≤2 mg/L | hsCRP >2 mg/L | | |
| Oral anticoagulation | | | | <0.01 |
| Yes | 7/476 (1.5) | 53/622 (8.5) | 5.99 (2.73-13.18) | |
| No | 50/1196 (4.2) | 155/2312 (6.7) | 1.62 (1.18-2.23) | |
| Severe or end-stage CKD | | | | 0.07 |
| Yes | 9/43 (20.9) | 43/199 (21.6) | 1.02 (0.50-2.09) | |
| No | 48/1629 (2.9) | 165/2735 (6.0) | 2.08 (1.51-2.87) | |
| Moderate or severe anemia | | | | 0.89 |
| Yes | 20/421 (4.8) | 95/1039 (9.1) | 1.96 (1.21-3.18) | |
| No | 37/1251 (3.0) | 113/1895 (6.0) | 2.05 (1.41-2.97) | |
| Spontaneous bleeding | | | | 0.44 |
| Yes | 3/59 (5.1) | 5/80 (6.3) | 1.22 (0.29-5.09) | |
| No | 54/1613 (3.3) | 203/2854 (7.1) | 2.16 (1.60-2.92) | |
| Moderate or severe thrombocytopenia | | | | 0.06 |
| Yes | 8/79 (10.1) | 15/154 (9.7) | 0.95 (0.40-2.25) | |
| No | 49/1593 (3.1) | 193/2780 (6.9) | 2.30 (1.68-3.15) | |
| Chronic bleeding diathesis | | | | 0.95 |
| Yes | 3/7 (42.9) | 0/6 (0.0) | - | |
| No | 54/1665 (3.2) | 208/2928 (7.1) | 2.23 (1.65-3.01) | |
| Active malignancy | | | | 0.73 |
| Yes | 2/55 (3.6) | 12/124 (9.7) | 2.74 (0.61-12.25) | |
| No | 55/1617 (3.4) | 196/2810 (7.0) | 2.09 (1.55-2.81) | |
| Previous cerebrovascular accident (major) | | | | 0.97 |
| Yes | 6/171 (3.5) | 15/211 (7.1) | 2.08 (0.81-5.35) | |
| No | 51/1501 (3.4) | 193/2723 (7.1) | 2.12 (1.56-2.89) | |
| Recent major surgery | | | | 0.97 |
| Yes | 0/12 (0.0) | 1/20 (5.0) | - | |
| No | 57/1660 (3.4) | 207/2914 (7.1) | 2.11 (1.57-2.82) | |
| Age ≥75 years | | | | 0.74 |
| Yes | 21/480 (4.4) | 83/868 (9.6) | 2.24 (1.39-3.62) | |
| No | 36/1192 (3.0) | 125/2066 (6.1) | 2.03 (1.40-2.94) | |
| Moderate CKD | | | | 0.46 |
| Yes | 18/284 (6.3) | 84/809 (10.4) | 1.66 (1.00-2.76) | |
| No | 39/1388 (2.8) | 124/2125 (5.8) | 2.11 (1.47-3.02) | |
| Mild anemia | | | | 0.73 |
| Yes | 19/659 (2.9) | 79/1225 (6.4) | 2.28 (1.38-3.77) | |
| No | 38/1013 (3.8) | 129/1709 (7.5) | 2.05 (1.42-2.94) | |
| Oral NSAIDs or steroids | | | | 0.50 |
| Yes | 3/90 (3.3) | 10/219 (4.6) | 1.38 (0.38-5.03) | |
| No | 54/1582 (3.4) | 198/2715 (7.3) | 2.18 (1.61-2.94) | |
| Previous cerebrovascular accident (minor) | | | | 0.20 |
| Yes | 10/371 (2.7) | 55/675 (8.1) | 3.09 (1.58-6.06) | |
| No | 47/1301 (3.6) | 153/2259 (6.8) | 1.91 (1.37-2.64) | |
| Prehospital statin use | | | | 0.59 |
| Yes | 14/560 (2.5) | 45/763 (5.9) | 2.40 (1.32-4.37) | |
| No | 43/1112 (3.9) | 163/2171 (7.5) | 1.97 (1.41-2.76) | |

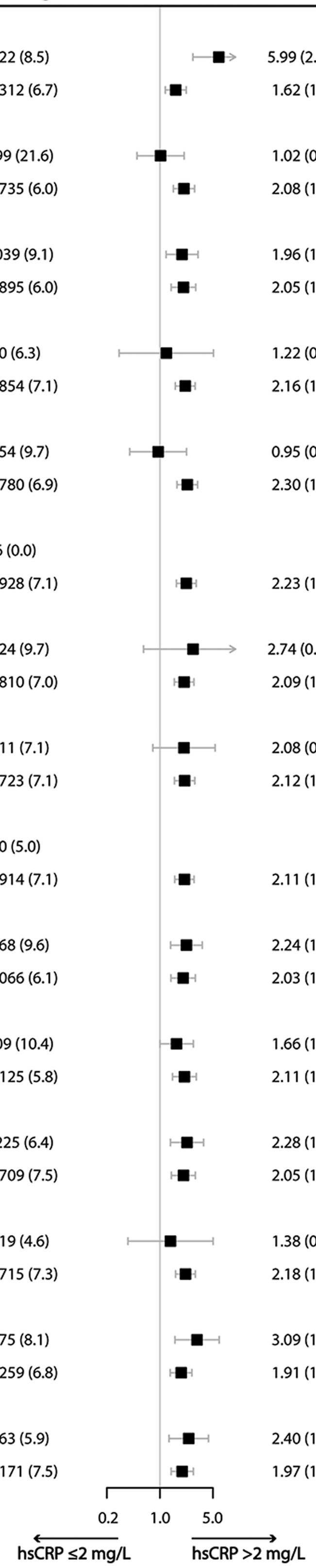


**Supplementary Fig. S3** Forest plot showing associations between high-sensitivity C-reactive protein (hsCRP) and all-cause death stratified by components of Academic Research Consortium–high bleeding risk (ARC-HBR) criteria and prehospital statin therapy.

 © 2025. The Author(s).

| | BARC types 2, 3, and 5 bleedings<br>*- Events/Total (%)* | | Hazard ratio (95%CI) | P for interaction |
|---|---|---|---|---|
| | hsCRP ≤2 mg/L | hsCRP >2 mg/L | | |
| Oral anticoagulation | | | | 0.07 |
| Yes | 33/476 (6.9) | 26/622 (4.2) | 0.62 (0.37-1.04) | |
| No | 63/1196 (5.3) | 129/2312 (5.6) | 1.08 (0.80-1.46) | |
| Severe or end-stage CKD | | | | 0.38 |
| Yes | 1/43 (2.3) | 11/199 (5.5) | 2.30 (0.30-17.83) | |
| No | 95/1629 (5.8) | 144/2735 (5.3) | 0.92 (0.71-1.19) | |
| Moderate or severe anemia | | | | 0.56 |
| Yes | 23/421 (5.5) | 47/1039 (4.5) | 0.84 (0.51-1.38) | |
| No | 73/1251 (5.8) | 108/1895 (5.7) | 1.00 (0.74-1.35) | |
| Spontaneous bleeding | | | | 0.52 |
| Yes | 5/59 (8.5) | 9/80 (11.3) | 1.36 (0.45-4.04) | |
| No | 91/1613 (5.6) | 146/2854 (5.1) | 0.93 (0.71-1.21) | |
| Moderate or severe thrombocytopenia | | | | 0.44 |
| Yes | 5/79 (6.3) | 13/154 (8.4) | 1.37 (0.49-3.84) | |
| No | 91/1593 (5.7) | 142/2780 (5.1) | 0.91 (0.70-1.19) | |
| Chronic bleeding diathesis | | | | 0.54 |
| Yes | 2/7 (28.6) | 1/6 (16.7) | 0.39 (0.04-4.40) | |
| No | 94/1665 (5.6) | 154/2928 (5.3) | 0.95 (0.74-1.23) | |
| Active malignancy | | | | 0.97 |
| Yes | 5/55 (9.1) | 10/124 (8.1) | 0.91 (0.31-2.67) | |
| No | 91/1617 (5.6) | 145/2810 (5.2) | 0.94 (0.72-1.22) | |
| Previous cerebrovascular accident (major) | | | | 0.55 |
| Yes | 6/171 (3.5) | 9/211 (4.3) | 1.27 (0.45-3.58) | |
| No | 90/1501 (6.0) | 146/2723 (5.4) | 0.91 (0.70-1.19) | |
| Recent major surgery | | | | 0.35 |
| Yes | 2/12 (16.7) | 1/20 (5.0) | 0.32 (0.03-3.51) | |
| No | 94/1660 (5.7) | 154/2914 (5.3) | 0.96 (0.74-1.23) | |
| Age ≥75 years | | | | 0.35 |
| Yes | 23/480 (4.8) | 46/868 (5.3) | 1.16 (0.70-1.91) | |
| No | 73/1192 (6.1) | 109/2066 (5.3) | 0.87 (0.65-1.18) | |
| Moderate CKD | | | | 0.48 |
| Yes | 17/284 (6.0) | 51/809 (6.3) | 1.09 (0.63-1.89) | |
| No | 79/1388 (5.7) | 104/2125 (4.9) | 0.87 (0.65-1.17) | |
| Mild anemia | | | | 0.14 |
| Yes | 35/659 (5.3) | 74/1225 (6.0) | 1.18 (0.79-1.76) | |
| No | 61/1013 (6.0) | 81/1709 (4.7) | 0.80 (0.57-1.11) | |
| Oral NSAIDs or steroids | | | | 0.66 |
| Yes | 3/90 (3.3) | 5/219 (2.3) | 0.69 (0.17-2.91) | |
| No | 93/1582 (5.9) | 150/2715 (5.5) | 0.96 (0.74-1.25) | |
| Previous cerebrovascular accident (minor) | | | | 0.85 |
| Yes | 19/371 (5.1) | 33/675 (4.9) | 0.99 (0.56-1.75) | |
| No | 77/1301 (5.9) | 122/2259 (5.4) | 0.93 (0.70-1.24) | |
| Prehospital statin use | | | | 0.53 |
| Yes | 44/560 (7.9) | 52/763 (6.8) | 0.88 (0.59-1.32) | |
| No | 52/1112 (4.7) | 103/2171 (4.7) | 1.04 (0.75-1.45) | |

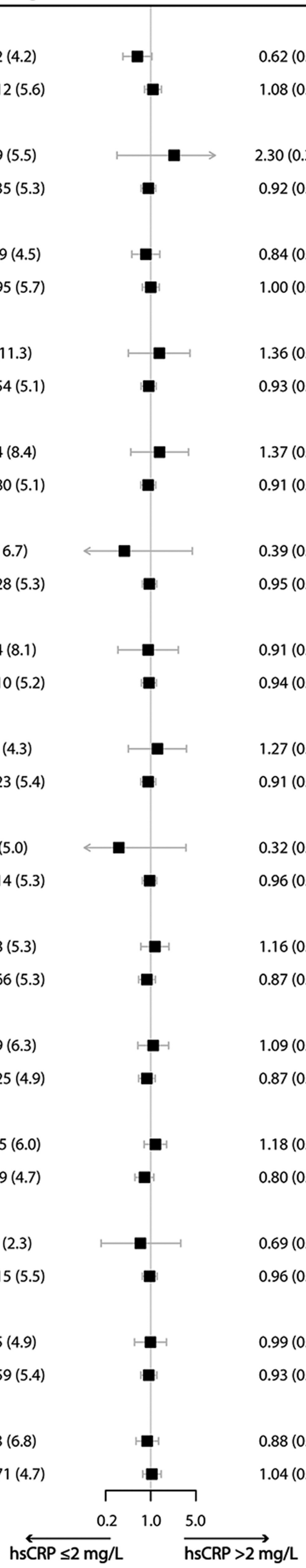


**Supplementary Fig. S4** Forest plot showing associations between high-sensitivity C-reactive protein (hsCRP) and Bleeding Academic Research Consortium (BARC) types 2, 3, and 5 bleedings stratified by Academic Research Consortium–high bleeding risk (ARC-HBR) criteria and prehospital statin therapy.

 © 2025. The Author(s).

**Supplementary Table S1** Adjusted association between HBR and adverse events at 1 year stratified by hsCRP

| | Low hsCRP (≤2 mg/L) (*N* = 6,618) | | | High hsCRP (>2mg/L) (*N* = 8,395) | | |
|---|---|---|---|---|---|---|
| | Non-HBR (*N* = 4,946) | HBR (*N* = 1,672) | *P*-value | Non-HBR (*N* = 5,461) | HBR (*N* = 2,934) | *P*-value |
| Ischemic events | 87 (1.8%) | 76 (4.5%) | <0.001 | 144 (2.6%) | 212 (7.2%) | <0.001 |
| Cardiac death | 44 (0.9%) | 46 (2.8%) | <0.001 | 77 (1.4%) | 162 (5.5%) | <0.001 |
| MI | 23 (0.5%) | 8 (0.5%) | 0.945 | 36 (0.7%) | 24 (0.8%) | 0.410 |
| Stroke | 24 (0.5%) | 25 (1.5%) | <0.001 | 38 (0.7%) | 30 (1.0%) | 0.111 |
| All-cause death | 49 (1.0%) | 57 (3.4%) | <0.001 | 94 (1.7%) | 208 (7.1%) | <0.001 |
| BARC types 2, 3, and 5 bleeding | 991 (20.0%) | 273 (16.3%) | 0.001 | 967 (17.7%) | 428 (14.6%) | <0.001 |
| BARC types 3 and 5 bleeding | 252 (5.1%) | 96 (5.7%) | 0.306 | 262 (4.8%) | 155 (5.3%) | 0.329 |

Abbreviations: BARC, Bleeding Academic Research Consortium; HBR, high bleeding risk; hsCRP, high-sensitivity C-reactive protein; MI, myocardial infarction.

**Supplementary Table S2** Relationship between HBR and different outcome events at different levels of hsCRP

| | Adjusted HR (95%CI) | *P*-value | |
|---|---|---|---|
| **Ischemic events** | | | |
| HBR | | | |
| hsCRP >1 mg/L | 1.65 (1.11–2.47) | 0.014 | hsCRP ≤1 mg/L as reference |
| hsCRP >2 mg/L | 1.20 (0.91–1.58) | 0.201 | hsCRP ≤2 mg/L as reference |
| hsCRP >3 mg/L | 1.37 (1.06–1.78) | 0.018 | hsCRP ≤3 mg/L as reference |
| Non-HBR | | | |
| hsCRP >1 mg/L | 1.53 (1.08–2.17) | 0.016 | hsCRP ≤1 mg/L as reference |
| hsCRP >2 mg/L | 1.34 (1.01–1.78) | 0.041 | hsCRP ≤2 mg/L as reference |
| hsCRP >3 mg/L | 1.63 (1.24–2.15) | 0.001 | hsCRP ≤3 mg/L as reference |
| **All-cause death** | | | |
| HBR | | | |
| hsCRP >1 mg/L | 2.16 (1.34–3.48) | 0.002 | hsCRP ≤1 mg/L as reference |
| hsCRP >2 mg/L | 1.50 (1.10–2.04) | 0.010 | hsCRP ≤2 mg/L as reference |
| hsCRP >3 mg/L | 1.58 (1.19–2.09) | 0.001 | hsCRP ≤3 mg/L as reference |
| Non-HBR | | | |
| hsCRP >1 mg/L | 1.79 (1.12–2.86) | 0.015 | hsCRP ≤1 mg/L as reference |
| hsCRP >2 mg/L | 1.49 (1.03–2.15) | 0.033 | hsCRP ≤2 mg/L as reference |
| hsCRP >3 mg/L | 1.71 (1.20–2.42) | 0.003 | hsCRP ≤3 mg/L as reference |
| **BARC types 2, 3, and 5 bleeding** | | | |
| HBR | | | |
| hsCRP >1 mg/L | 1.29 (0.91–1.82) | 0.146 | hsCRP ≤1 mg/L as reference |
| hsCRP >2 mg/L | 0.96 (0.73–1.26) | 0.758 | hsCRP ≤2 mg/L as reference |
| hsCRP >3 mg/L | 0.92 (0.71–1.21) | 0.563 | hsCRP ≤3 mg/L as reference |
| Non-HBR | | | |
| hsCRP >1 mg/L | 1.07 (0.87–1.31) | 0.523 | hsCRP ≤1 mg/L as reference |
| hsCRP >2 mg/L | 0.95 (0.80–1.15) | 0.615 | hsCRP ≤2 mg/L as reference |
| hsCRP >3 mg/L | 0.86 (0.71–1.04) | 0.111 | hsCRP ≤3 mg/L as reference |

(*Continued*)

 © 2025. The Author(s).

**Supplementary Table S2** (*Continued*)

| | | | |
|---|---|---|---|
| **BARC types 3 and 5 bleeding** | | | |
| HBR | | | |
| hsCRP >1 mg/L | 0.98 (0.52–1.84) | 0.950 | hsCRP ≤1 mg/L as reference |
| hsCRP >2 mg/L | 0.89 (0.53–1.51) | 0.673 | hsCRP ≤2 mg/L as reference |
| hsCRP >3 mg/L | 1.14 (0.69–1.88) | 0.622 | hsCRP ≤3 mg/L as reference |
| Non-HBR | | | |
| hsCRP >1 mg/L | 1.14 (0.74–1.76) | 0.539 | hsCRP ≤1 mg/L as reference |
| hsCRP >2 mg/L | 1.02 (0.69–1.49) | 0.939 | hsCRP ≤2 mg/L as reference |
| hsCRP >3 mg/L | 0.96 (0.65–1.42) | 0.840 | hsCRP ≤3 mg/L as reference |

Abbreviations: BARC, Bleeding Academic Research Consortium; HBR, high bleeding risk; hsCRP, high-sensitivity C-reactive protein.
Note: Model adjusted for age, sex, hypertension, diabetes, previous MI, previous stroke, previous PCI, PAD, smoking status, presentation, eGFR, anemia, and medical treatment at discharge.

 © 2025. The Author(s).

**Supplementary Table S3** Multivariable competing risk analysis and multivariable Cox regression analysis for outcomes stratified by HBR and hsCRP at 1 year

| | Non HBR ($N=10,407$) | | | | HBR ($N=4,606$) | | | |
|---|---|---|---|---|---|---|---|---|
| | Cox proportional hazards model | | Competing risks model | | Cox proportional hazards model | | Competing risks model | |
| | Adjusted HR (95%CI) | *P*-value | Adjusted HR (95%CI) | *P*-value | Adjusted HR (95%CI) | *P*-value | Adjusted HR (95%CI) | *P*-value |
| Ischemic events | 1.34 (1.01–1.78) | 0.041 | 1.34 (1.001–1.79) | 0.049 | 1.20 (0.91–1.58) | 0.201 | 1.21 (0.91–1.61) | 0.200 |
| Cardiac death | 1.29 (0.87–1.92) | 0.200 | 1.29 (0.86–1.94) | 0.219 | 1.34 (0.95–1.89) | 0.097 | 1.36 (0.94–1.96) | 0.099 |
| MI | 1.42 (0.82–2.47) | 0.214 | 1.42 (0.79–2.55) | 0.243 | 1.35 (0.57–3.20) | 0.492 | 1.36 (0.60–3.07) | 0.464 |
| Stroke | 1.45 (0.84–2.50) | 0.180 | 1.45 (0.83–2.55) | 0.197 | 0.80 (0.46–1.40) | 0.432 | 0.79 (0.44–1.40) | 0.418 |
| All-cause death | 1.49 (1.03–2.15) | 0.033 | – | – | 1.50 (1.10–2.04) | 0.010 | – | – |
| BARC types 2,3, and 5 bleeding | 0.95 (0.80–1.15) | 0.615 | 0.95 (0.79–1.15) | 0.605 | 0.96 (0.73–1.26) | 0.758 | 0.95 (0.73–1.24) | 0.714 |
| BARC types 3 and 5 bleeding | 1.02 (0.69–1.49) | 0.939 | 1.01 (0.68–1.50) | 0.952 | 0.89 (0.53–1.51) | 0.673 | 0.90 (0.55–1.47) | 0.668 |

Abbreviations: BARC, Bleeding Academic Research Consortium; HBR, high bleeding risk; hsCRP, high-sensitivity C-reactive protein.
Note: Model adjusted for age, sex, hypertension, diabetes, previous MI, previous stroke, previous PCI, PAD, smoking status, presentation, eGFR, anemia, and medical treatment at discharge.

 © 2025. The Author(s).

**Supplementary Table S4** Outcomes of individuals stratified by PRECISE-DAPT score

| | PRECISE-DAPT score | | HR (95%CI) | *P*-value |
|---|---|---|---|---|
| | <25 (*N* = 13,022) | ≥25 (*N* = 1,989) | | |
| Ischemic events | 330 (2.5%) | 189 (9.5%) | 3.96 (3.31–4.74) | <0.001 |
| Cardiac death | 180 (1.4%) | 149 (7.5%) | 5.67 (4.56–7.04) | <0.001 |
| MI | 68 (0.5%) | 23 (1.2%) | 2.37 (1.48–3.81) | <0.001 |
| Stroke | 96 (0.7%) | 21 (1.1%) | 1.53 (0.95–2.45) | 0.079 |
| All-cause death | 222 (1.7%) | 186 (9.4%) | 5.76 (4.74–7.00) | <0.001 |
| BARC types 2, 3, and 5 bleeding | 656 (5.0%) | 109 (5.5%) | 1.15 (0.94–1.41) | 0.173 |
| BARC types 3 and 5 bleeding | 151 (1.2%) | 37 (1.9%) | 1.71 (1.19–2.45) | 0.004 |

Abbreviations: BARC, Bleeding Academic Research Consortium; HBR, high bleeding risk; hsCRP, high-sensitivity C-reactive protein.
Note: Model adjusted for age, sex, hypertension, diabetes, previous MI, previous stroke, previous PCI, PAD, smoking status, presentation, eGFR, anemia, and medical treatment at discharge.

 © 2025. The Author(s).

**Supplementary Table S5** Outcomes of individuals stratified by PRECISE-DAPT score and hsCRP at 1 year

| | PRECISE-DAPT <25 ($N = 13{,}022$) | | | | PRECISE-DAPT ≥25 ($N = 1{,}989$) | | | | P for interaction |
|---|---|---|---|---|---|---|---|---|---|
| | Low hsCRP (≤2 mg/L) ($N = 6{,}085$) | High hsCRP (>2 mg/L) ($N = 6{,}937$) | Adjusted HR (95%CI) | *P*-value | Low hsCRP (≤2 mg/L) ($N = 531$) | High hsCRP (>2 mg/L) ($N = 1{,}458$) | Adjusted HR (95%CI) | *P*-value | |
| Ischemic events | 124 (2.0%) | 206 (3.0%) | 1.33 (1.05–1.68) | 0.019 | 39 (7.3%) | 150 (10.3%) | 1.14 (0.79–1.65) | 0.476 | 0.879 |
| Cardiac death | 62 (1.0%) | 118 (1.7%) | 1.40 (1.01–1.94) | 0.042 | 28 (5.3%) | 121 (8.3%) | 1.23 (0.80–1.90) | 0.339 | 0.811 |
| MI | 28 (0.5%) | 40 (0.6%) | 1.26 (0.75–2.11) | 0.376 | 3 (0.6%) | 20 (1.4%) | 2.24 (0.64–7.90) | 0.209 | 0.299 |
| Stroke | 40 (0.7%) | 56 (0.8%) | 1.23 (0.80–1.89) | 0.344 | 9 (1.7%) | 12 (0.8%) | 0.63 (0.25–1.61) | 0.337 | 0.078 |
| All-cause death | 75 (1.2%) | 147 (2.1%) | 1.51 (1.13–2.03) | 0.006 | 31 (5.8%) | 155 (10.6%) | 1.50 (1.00–2.24) | 0.049 | 0.767 |
| BARC types 2, 3, and 5 bleeding | 318 (5.2%) | 338 (4.9%) | 0.96 (0.82–1.13) | 0.662 | 30 (5.6%) | 79 (5.4%) | 0.98 (0.63–1.52) | 0.929 | 0.934 |
| BARC types 3 and 5 bleeding | 72 (1.2%) | 79 (1.1%) | 0.93 (0.67–1.31) | 0.685 | 8 (1.5%) | 29 (2.0%) | 1.17 (0.51–2.69) | 0.714 | 0.552 |

Abbreviations: BARC, Bleeding Academic Research Consortium; HBR, high bleeding risk; hsCRP, high-sensitivity C-reactive protein.
Note: Model adjusted for age, sex, hypertension, diabetes, previous MI, previous stroke, previous PCI, PAD, smoking status, presentation, eGFR, anemia, and medical treatment at discharge.

 © 2025. The Author(s).
